# Life History Traits of Sperm Whales *Physeter macrocephalus* Linnaeus, 1758 Stranded along Italian Coasts (Cetartiodactyla: Physeteridae)

**DOI:** 10.3390/ani13010079

**Published:** 2022-12-25

**Authors:** Nicola Maio, Tatiana Fioravanti, Lucrezia Latini, Agnese Petraccioli, Marcello Mezzasalma, Bruno Cozzi, Sandro Mazzariol, Michela Podestà, Gianni Insacco, Francesco Pollaro, Giuseppe Lucifora, Ida Ferrandino, Nicola Zizzo, Filippo Spadola, Fulvio Garibaldi, Fabio Maria Guarino, Andrea Splendiani, Vincenzo Caputo Barucchi

**Affiliations:** 1 Dipartimento di Biologia, Università degli Studi di Napoli Federico II, Via Cinthia 26, 80126 Napoli, Italy; 2 Dipartimento di Scienze della Vita e dell’Ambiente, Università Politecnica delle Marche, Via Brecce Bianche, 60131 Ancona, Italy; 3 Dipartimento di Biologia, Ecologia e Scienze della Terra, Università della Calabria, Via P. Bucci 4/B, 87036 Rende, Italy; 4 Dipartimento di Biomedicina Comparata e Alimentazione, Università degli Studi di Padova, Viale dell’Università 16, 35020 Padova, Italy; 5 Museo Civico di Storia Naturale di Milano, Sezione di Zoologia dei Vertebrati, Corso Venezia 55, 20121 Milano, Italy; 6 Museo Civico di Storia Naturale di Comiso, via degli Studi 9, 97013 Ragusa, Italy; 7 Centro Studi Ecosistemi Mediterranei, Via Caracciolo, 84060 Pollica, Italy; 8 Istituto Zooprofilattico Sperimentale del Mezzogiorno, 80055 Portici, Italy; 9 Dipartimento di Medicina Veterinaria, Università degli Studi di Bari Aldo Moro, Piazza Umberto I, 70121 Bari, Italy; 10 Museo della Fauna, Dipartimento di Scienze Veterinarie, Università degli Studi di Messina, 98168 Messina, Italy; 11 DISTAV, Dipartimento di Scienze della Terra, dell’Ambiente e della Vita Università degli Studi di Genova, Corso Europa 26, 16132 Genova, Italy

**Keywords:** cetacea, odontocetes, Italian sea, geographic origin, age estimation, age at sexual maturity

## Abstract

**Simple Summary:**

Several life-history traits of Mediterranean sperm whales are little explored. We present new original data on the relationships between age and body length and age at maturity of individuals stranded along the Italian coast. We found that Mediterranean male sperm whales attain sexual maturity at 10 years and for the same body length class are older than Atlantic ones. Our finding of a Mediterranean pregnant female of only 6.5 m of body length and an assessed age of 24–26 years is particularly noteworthy, as the females of this species reach sexual maturity at about 9 meters total length and 9 years of age in other marine areas. Two different hypotheses can be advanced to explain this difference in size, based on either ecological or genetic factors. The first is based on a lesser energy intake of Mediterranean individuals compared to Atlantic ones, mostly due to a smaller prey size and less trophic conditions. The second hypothesis concerns a low genetic diversity and mating among closely related individuals. Considering its low genetic diversity, the Mediterranean sperm whale population should be the target of focused conservation efforts as it might be relatively less responsive to environmental changes.

**Abstract:**

We investigated the relationship between age and body length, and age at sexual maturity of *Physeter macrocephalus* individuals stranded along the Italian coast. Our molecular analysis shows that all our samples belong to the C.001.002 haplotype, shared between Atlantic and Mediterranean populations. We show that males attain sexual maturity at 10 years, similar to those from other marine areas. However, considering the same body length class, Mediterranean males are older than Atlantic ones. Our finding of a Mediterranean pregnant female of only 6.5 m in length and an assessed age of 24–26 years is particularly noteworthy, considering that females reach sexual maturity at about 9 years and 9 m of total length in other regions. Comparing our results with the literature data, we highlight the positive correlation between lifespan, adult body length and weight of males from the Mediterranean and Atlantic Ocean. Regardless of whether the relatively small size of Mediterranean specimens is a consequence of an inbreeding depression or an adaptation to less favorable trophic conditions, we recommend to closely monitor this population from a conservation perspective. In fact, its low genetic diversity likely corresponds to a relatively limited ability to respond to environmental changes compared with other populations.

## 1. Introduction

The sperm whale, *Physeter macrocephalus* Linnaeus, 1758, is an odontocete cetacean having one of the widest global distributions among marine mammal species. It is found in all deep ocean waters, from the equator to Arctic and Antarctic regions (downloaded on 18 June 2022 from https://www.fisheries.noaa.gov/species/sperm-whale). In the Mediterranean Sea, *P. macrocephalus* is considered a regular inhabitant, but is much more abundant in the Western Basin than in the Eastern one [1,2,3,4,5,6]. Concerning the waters surrounding the Italian coasts, this species is more often found in the Tyrrhenian Sea (especially in the Ligurian Sea and west of Corsica and Sardinia) and in the Ionian Sea (especially along the Hellenic Trench), while it is less frequently observed in the Adriatic Sea where the habitat is less favourable [3,7].

The sperm whale is the largest of the toothed whales, with males reaching up to 21 m in length [8]. It is a typical K-selected species, with a biological cycle associated with stable environmental conditions, a slow attainment of sexual maturity (typically 10–13 years, exceptionally 7 years in females, 18–21 years in males, high longevity (up to 80 years) [9,10,11,12], few offspring (the female is able to give birth to a single calf every 4–5 years, and subsequently might be expected to produce only 4–5 offspring throughout her lifetime), and high amount of parental care (lactation period lasts about 2 years, sometimes up to 5 years, and follows a gestation period of 14–16 months) [10].

Most of the knowledge on the biology of this species comes from studies on animals inhabiting the waters of the Pacific [11,12,13,14], Atlantic [15,16] and Indian ocean [9,10,17]. By contrast, the Mediterranean sperm whales are still poorly known and information on several aspects of their life-history traits, including body growth rate and age at sexual maturity, is scarce and limited to a few studies [7,18,19]. The acquisition of such knowledge is even more important considering that the Mediterranean sperm whale population shows a very low genetic variability compared to individuals from the Atlantic [20]. This is probably due to a recent population expansion and the diffusion in the Mediterranean Basin of a single matriline during the last glacial maximum (LGM, about 20,000 years ago) [21]. Similar to other cetacean species, dead sperm whales are also found stranded along the Italian coast and may provide valuable samples to elucidate many aspects of the biology of the species in the Mediterranean Sea [22]. 

Here, we used a multidisciplinary approach combining molecular, morphological and statistical analyses as integrative methodologies are better suited to describe different life history traits and their possible correlations [23,24,25,26,27].

In particular, this study was conducted on Mediterranean sperm whales stranded along Italian coasts in order to: (i) establish their putative geographic origin based on mitochondrial DNA (mtDNA) haplotypes; (ii) estimate the individual age by counting the growth layer groups (GLGs) in the teeth; (iii) analyse the age-length relationship; (iv) estimate the age at sexual maturity by matching individual age assessed by GLGs count and reproductive status.

## 2. Materials and Methods

### 2.1. Study Sample

Sperm whale samples used in this study (*n* = 16) and the relative data on their stranding place, date, sex, and total body length of the animals are listed in Table 1.

### 2.2. Genetic Analysis

Samples of muscle tissue from ten *P. macrocephalus* individuals (2330A, 549, 400, 154159, 456, 45486, 463, 465, 466, 467) (Table 1) were collected from the MMTB and stored in 70% ethanol until genetic analyses were performed. DNA was extracted using a standard phenol/chloroform protocol [28]. The quantity and purity of the extracted DNA were determined with a NanoDrop-ND1000 spectrophotometer (NanoDrop Technologies, Inc., Wilmington, DE, USA)).

A fragment of the mitochondrial DNA control region (mtDNA CR), comprising the 619 bp sequence previously obtained by Alexander et al. [20]) was amplified by polymerase chain reaction (PCR) using a primer pairs (PmCR_F: 5′-GCACCCAAAGCTGAAATTCT-3′, PmCR_R: 5′-ACACACAGGTCCGGCTAAGA-3′) specifically designed on Primer3Plus software [29]. PCR amplifications were carried out in a reaction volume of 25 μL containing 5 μL of 5× MyTaq™ Reaction Buffer (BioLine, London, UK), 2.5 μL of F + R primer solution [5 μM], 0.3 μL of MyTaq™ DNA Polymerase (BioLine), 3 μL of DNA template [~40 ng/μL] and 14.2 μL of ddH2O. The thermal cycle profile consisted of an initial denaturation at 95 °C for 5 min followed by 30 cycles of denaturation at 95 °C for 45 s, annealing at 55 °C for 45 s, extension at 72 °C for 90 s, and a final extension step at 72 °C for 7 min. The amplification success was checked on a 2% agarose gel stained with GelRed™ (Biotium) and PCR products were sent to the BMR Genomics (Padova, Italy), where they were purified by exoSAP-IT™ (USB Corp., Cleveland, OH, USA) and Sanger sequenced in both directions on an ABIPRISM 3730XL automated sequencer (Applied Biosystems, Tokyo, Japan).

All mtDNA CR sequences obtained from our samples (619 bp) were aligned using ClustalW with those of the 52 haplotypes already known for *P. macrocephalus* (GenBank Accession numbers KU719571-KU719622) and combined in Alexander et al. [20]. The alignment was verified on BioEdit [30] and diagnostic sites were identified to assign our sequences to a specific haplotype, and consequently to a geographic region, on the basis of information obtained from the literature about the worldwide distribution of mtDNA diversity in *P. macrocephalus* [20,31,32,33]. In order to allow the comparison of our sequences with those of haplotypes already found in Mediterranean individuals, only the first 394 bp of the mtDNA CR were actually considered [20,32]

### 2.3. Age Determination

Mandibular teeth of eight specimens (GP-1, 549, 400, 154159, 456, 45486, 465, 12988 (Table 1) were used for age estimation. Of three specimens (172, 173 and 174), the age was already assessed by Mazzariol et al. [7] while the teeth of the remaining five specimens were not available in tissue banks. Teeth were processed according to Evans and Robertson [34]. Namely, each tooth was bisected along the sagittal plane using a low-speed diamond metallographic saw. The cut surface of both halves of each tooth was polished by hand using a wet fine-grade sandpaper (150 and 320 grit). Both halves were then placed in a bath of 15% formic acid, with the cut and polished surface down, at room temperature until a clear and complete etched surface was produced (at least three hours). Afterward, each tooth half was placed under running tap water (3 min), then removed and placed in a bath of acetone (3 min); the last two steps was repeated several times until the GLGs were clearly visible. The etched surface of each tooth was then examined using a Leica EZ4 stereo microscope (Leica Microsystems GmbH, Wetzlar, Germany) under reflected light and equipped with a digital camera. Different portions of tooth sections were acquired at high magnification by a digital camera and successively stitched together into a single image using the free download program AutoStitch64. The count of GLGs was performed independently by two researchers (FMG and NM) and without prior knowledge of the total length and sex of the specimens. In the case of discrepancies in the GLG count, the etched surface of the tooth was read again until a final consensus was reached [35,36].

### 2.4. Reproductive Status and Sexual Maturity

Testis samples of five specimens (172, 173, 174, 456, 45486) (Table 1) were available for evaluating the state of maturity of the males. Small pieces of testis were fixed in neutral buffered formalin and embedded in paraffin following standard protocols. They were then sectioned at 7 μm thick using a semi-automated rotary microtome. The sections were stained with Mallory Trichrome (Bioptica, Milano, Italy) and observed under a Motic BA340 light microscope (Motic Deutschland GmbH, Wetzlar, Germany) equipped with a digital camera. The presence of different germ cell stages in the seminiferous tubules was assessed and only animals in advanced spermatogenesis, including spermatids and sperms, were considered mature according to Honh et al. [37]. Concerning the reproductive status of females, we considered if they were pregnant or not pregnant, lactating with scalf or not lactating, as ovarian samples were not available.

## 3. Results

### 3.1. Genetic Analysis

DNA extraction, amplification and sequencing were successfully performed for all the ten *P. macrocephalus* individuals sampled. All the sequences obtained were deposited in GenBank (Accession numbers OQ060609–OQ060618)The multiple alignment including our sequences and those of mtDNA CR haplotypes previously described [20] highlights the presence of 34 diagnostic sites and shows that all our samples belong to the C.001.002 haplotype (Table 2).

### 3.2. Age Estimation and Body Length

The age estimation of animals examined in this study (*n* = 8) is given in Table 3.

**Figure 1:**
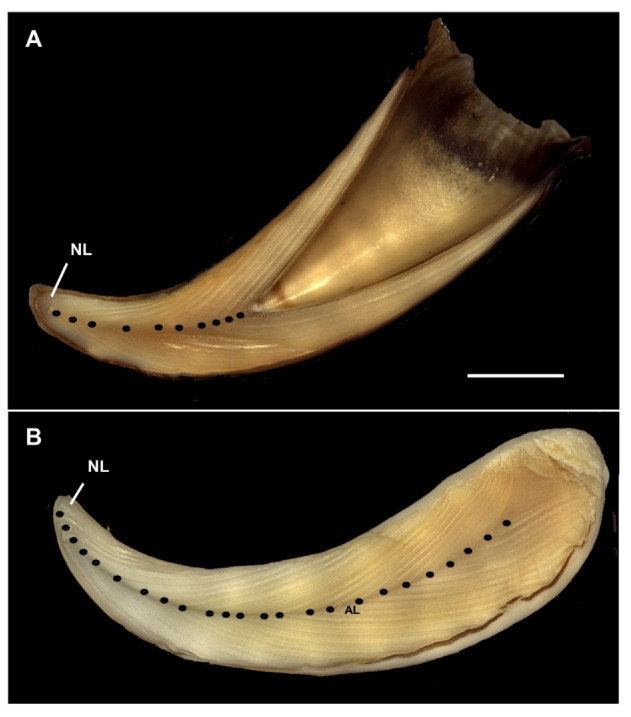
Representative composite photographs of sperm whale longitudinal, acid-etched, tooth sections. (**A**) ID 456, male, with number of GLGs estimated as 10. (**B**) ID 549, female, with number of GLGs estimated as 24–26. The filled circle indicates the start of each GLG. NL: neonatal line. AL: accessory line. Scale bar:1.5 cm in (**A**) e 1.4 cm in (**B**).

There was no discordance in GLG counting intra and inter reader with exception of four animals (ID 549, 400, 45486 and 12988) where the presence of accessory layers (see [38]) confounded the GLG reading (Figure 1). The youngest and even the smallest (unsexed) individual (ID 154159, 6.10 m of TBL) was estimated as 3 years old. The oldest and also biggest individual was a male (ID 45486) 12.16 m of TBL, with an estimated age of 40–42 years old. The ID 549 female, 6.50 m of TBL, was a pregnant individual with an estimated age of 24–26 years.

**Figure 2:**
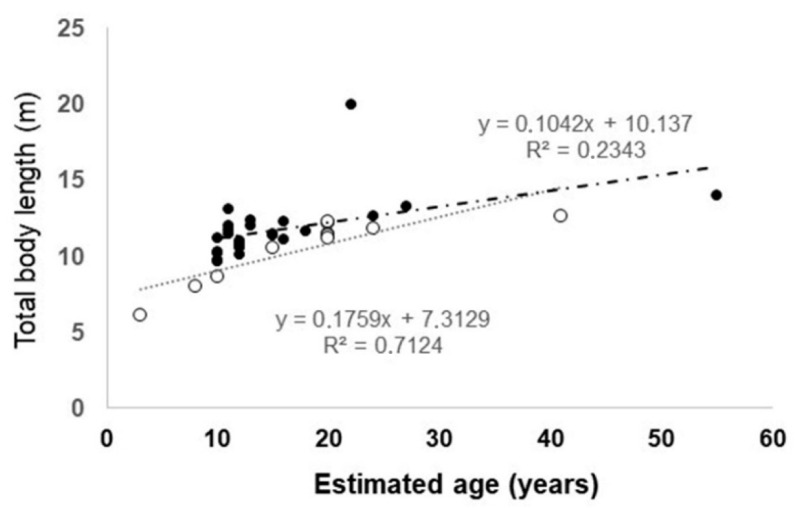
Relationship between age and total body length in Mediterranean (open circle) and Atlantic (filled circle) male sperm whales. Linear regression equations are also showed.

The relationship between age and TBL in Mediterranean and Atlantic sperm whales is shown in Figure 2, which is based on the original data of the present study combined with available bibliographical data by Mazzariol et al. [7], Borrell et al. [15] and IJsseldijk et al. [16]. Since the female sample was too small, age/TBL relationship was statistically estimated only in males. There was a significant positive correlation between age and body length both in Mediterranean (Pearson’s correlation coefficient r = 0.843, df = 9, *p* < 0.01) and in Atlantic sperm whales (r = 0.483, df = 23), *p* < 0.05) but the slope of the regression line was significantly different between Mediterranean and Atlantic animals (F_1,29_ = 5.26, *p* = 0.02), indicating that for a given TBL Mediterranean sperm whales were older than Atlantic sperm whales.

### 3.3. Reproductive Status

**Figure 3:**
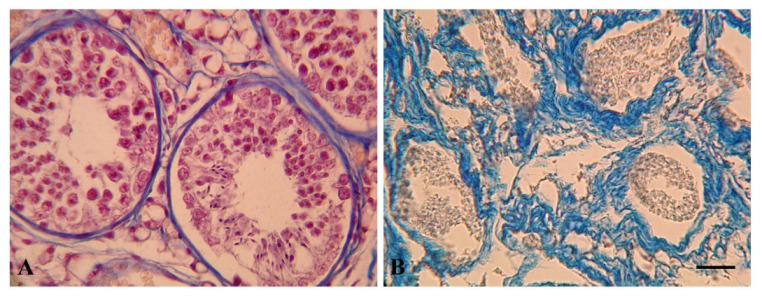
Histological sections of testis of *P. macrocephalus*. (**A**) ID 174: seminiferous tubules were large with an open lumen and a complex seminiferous epithelium where spermatogonia, spermatocytes and spermatids are recognizable. (**B**) ID 456: seminiferous tubules were in advanced autolytic state and surrounded by abundant interstitial tissue. Scale bar: 30 µm.

All the testicular samples examined (*n* = 5) were in a variable state of autolysis with structural alterations due to a poor state of conservation, which interfered with the identification of the spermatogenic stages in some specimens. The animals ID 172, 173, 174, 45486 had seminiferous tubules with a large lumen and a developed epithelium with cellular elements ascribable to stages of advanced spermatogenesis, including spermatids and sperms (Figure 3). For the individual ID 456 it was not possible to interpret the spermatogenic stage.

Among the five females examined, three (ID 549, 400 and 463) were pregnant, one (ID 12988) was lactating with calf, and one (ID 465) without evident characteristics about the attainment of sexual maturity.

## 4. Discussion

In *P. macrocephalus*, the genetic diversity of the mtDNA CR was described in the Mediterranean Sea mainly using a shorter sequence (394 bp) than the one analysed in this study [20,31,32]. The shorter sequence does not allow to distinguish between C.001.001, C.001.002 and C.002.001 haplotypes, which are therefore defined as a single haplotype called “C” (Table 2). The haplotype C is one of the most common haplotypes described in *P. macrocephalus*. It was observed in individuals from Atlantic, Indian and Pacific Ocean and, in the Mediterranean Sea, it is the unique haplotype described so far [20,32]. Thus, although the observation of haplotype C does not in itself represent forensic evidence of the Mediterranean origin of the samples examined in this study, the fact that they are all referable to the same haplotype is a typical signature of the Mediterranean sperm whale, as already documented in previous studies (e.g., [7]). This is probably due to a recent population expansion and the diffusion in the Mediterranean Basin of a single matriline during the LGM [21].

In cetaceans, there is a positive correlation between lifespan and adult body length, and adult body weight so that as a rule the largest animals are usually also the oldest [39]. Our study revealed that this correlation is true also for male sperm whales from the Mediterranean Sea and North Atlantic Ocean. Furthermore, we showed that at the same body size class Mediterranean male sperm whales are older than specimens from the Atlantic.

The age and body length at sexual maturity of the Mediterranean male sperm whales from this study (10 years and 10.5 m, respectively) are similar to those reported for this species in the north-western Pacific (9 years and 9.15 m, in Nishiwaki et al. [11]), and in the southwestern Indian Ocean (8–9 years and 10 m, in Best [10]). Regarding the northwestern Atlantic, in the study by Ijsseldik et al. [16] male sperm whales with body length ranging from 9.6 to 14.7 m and age between 10 and 16 years were reported as immature. However, although male sperm whales can produce sperms even when they are 10 m in body length, their fertility potential may be markedly lower than that of larger males [9,10]. Furthermore, as in other odontocetes [40,41], after attaining sexual maturity, male sperm whales typically continue to grow for several years until physical maturity is reached [10] In fact, although a juvenile male is physiologically capable of reproducing, he is rarely able to mate successfully with a female or compete with dominant males until he is older and larger [41]. For female sperm whales, we were not able to analyse the relationship between age and body length due to a small sample size. However, one of the most intriguing results was that a female of just 6.5 m in body length (ID 549) was pregnant and with an estimated age of 22–24 years. To the best of our knowledge, this individual represents the smallest pregnant female of *P. macrocephalus* recorded so far. In addition, the body length of this pregnant female is markedly smaller than that reported in literature as the minimum body length at sexual maturity for females from other marine areas (about 8,9 m for Pacific Ocean, Nishiwaki et al. [11]; 8–9 m for waters off South African coasts, in Best, [10]. Furthermore, the female ID 549 was much older than female sperm whale of comparable body length inhabiting marine areas different from Mediterranean Sea. Interestingly, the other sexually mature female specimens of sperm whale analysed in this study (ID 12988, 10 m of TBL and 38–40 years old) and those stranded along the Adriatic coast reported in Mazzariol et al. [19] (ID 1, 8.95 m of TBL and 31–32 years old; ID 2, 8.38 m and 21 years old) are also small-sized but quite long-lived when compared to females from other marine areas.

Best et al. [42] shown that there is a strong geographical variation in body size of adult sperm whales and, as a rule, individuals collected in tropical waters are significantly smaller than those in temperate regions. The factors that could produce such diversity in size include a different prey availability, which directly influences energy intakes and growth rates, or even that different populations have differing prey preferences that they occupy different geographical regions. For example, there are indications of declines in the size of individual teuthids from high to low latitudes [43,44], and some data on declining blubber thickness in female sperm whales from high to low latitudes [45]. Concerning the Mediterranean Sea, its oligotrophic condition (e.g. [46]) could explain a lower energy supply of sperm whale prey compared to the Atlantic Ocean, and this might be at the base of the low growth rate compared to larger animals inhabiting the Atlantic Ocean. In fact, in the Mediterranean Sea, the eating habits of the sperm whale are mainly based on the medium-sized cephalopod *Histioteuthis bonnellii* (e.g., [7,47]), compared to the much bigger *Architeuthis* whose sporadic presence in the Mediterranean basin has been reported only recently [48]. The "Levantine nanism" in bottlenose dolphins could support this hypothesis. In fact, Eastern Mediterranean dolphins are characterized by a smaller size compared to other Mediterranean populations because the lower primary production of their environment seems to favour an anticipation of the time of sexual maturity, which corresponds to an earlier stunting [49]. However, in our case, the difference in size could be due more to a slower overall growth rate than to an early reaching of sexual maturity. In fact, our data suggest that, at least in males, both the Atlantic and Mediterranean populations reach reproductive maturity substantially at the same body size. On the other hand, the well-known case of the Pacific killer whale [50] strengthens the hypothesis that food quality is the main factor that affects the size of individuals belonging to different populations. Indeed, among the three different-size ecotypes described on the base of habitat and prey preference, the ecotype C includes the smallest individuals eating fish, compared to the larger ecotypes A and B whose alimentation is mainly oriented on large and energy-rich marine mammals.

On the other hand, a different scenario could be suggested to explain the small size of the Mediterranean sperm whale population. Indeed, sperm whales inhabiting the Mediterranean Sea are characterized by low genetic variation (e.g., [7,21]), also confirmed by our data showing the presence of a single mtDNA CR haplotype (C.001.002) for all the examined animals. This is likely the result of a founder event linked to the recent colonization of the Mediterranean Sea, about 20,000 years ago, when a “lost tribe” or an extended “lobe” of the large North Atlantic sperm whale population became isolated in this basin [51]. The limited gene flow through the Strait of Gibraltar [52] and the small population size, where most or all mates are closely related, might have promoted inbreeding and inbreeding depression favouring the segregation of deleterious recessive alleles such as those related to dwarfism (see [53]), as the very small size (only 6.5 m, see above) of a pregnant female strongly suggest. A case very similar to that of Mediterranean sperm whale was described for the Australian pygmy blue whale (*Balaenoptera musculus brevicauda*), which is characterized by a low genetic variability established in consequence of a founder effect from Antarctic blue whales around the LGM [54]. However, in this case the authors hypothesized that after being founded, the Australian pygmy blue whales became phenotypically (smaller body length) and behaviourally (song type) distinct from Antarctic blue whales. This suggests that the Australian population not only became genetically different through the stochastic processes of genetic drift, but also through natural selection primarily driven by adaptation to the reduced biological productivity of their new habitat [54].

## 5. Conclusions

We conducted a multidisciplinary study on sperm whales stranded along the Italian coasts in order to increase the knowledge on the poorly known biology of the individuals inhabiting the Mediterranean Basin, as performed also on other species [4,5,55]. Comparing our results with those available from the literature, we highlight the occurrence of a positive correlation between lifespan and adult body length in *P. macrocephalus* males from the Mediterranean Sea and North Atlantic Ocean. We also showed that males attain sexual maturity at 10 years and for the same body length class Mediterranean male sperm whales are older than Atlantic ones. Our finding of a Mediterranean pregnant female of only 6.5 m of body length and an assessed age of 24–26 years is particularly noteworthy, representing smallest pregnant female of *P. macrocephalus* recorded so far. Regardless if the small size of Mediterranean sperm whales is a consequence of inbreeding depression or adaptation to the oligotrophic condition of the Mediterranean Basin, it is appropriate to closely monitor this population in a conservation perspective, as the naturally low genetic diversity means they likely have a lower ability to respond to today’s changing environment compared with other sperm whale populations, with the risk of hesitating in an extinction vortex (*sensu* [56]).

## Figures and Tables

**Table 1:** Specimens of *P. macrocephalus* used in this study. F: female; Juv: juvenile; M: male; TBL: total body length.

ID	Stranding Place	Stranding Date	Sex	TBL (m)
Ph1	Forio (Napoli)	23 April 1770	M	10
GP-1	Castellabate (Salerno)	1980	M juv	7.2
172	Cagnano Varano (Foggia)	10 December 2009	M	11.2
173	Cagnano Varano (Foggia)	10 December 2009	M	12.14
174	Cagnano Varano (Foggia)	10 December 2009	M	10.50
2330A	Polignano a Mare (Bari)	29 September 2014	juv	8
549	Acquedolci (Messina)	3 June 2015	F	6.50
400	Bagheria (Palermo)	12 October 2016	F	8.4
154159	Parghelia (Vibo Valentia)	26 December 2017	juv	6.10
456	Forio (Napoli)	26 December 2018	M juv	8.6
45486	San Lucido (Cosenza)	3 April 2018	M	12.16
463	Porto Cervo, Arzachena (Sassari)	28 March 2019	F	8
465	Capo Plaia, Cefalù (Palermo)	16 May 2019	F	6.26
466	Gioiosa Marea (Messina)	21 May 2019	M	5.35
467	Acqua dei Corsari (Palermo)	19 May 2019	M	8.5
12988	Near to Palmarola (Latina)	19 June 2019	F	10

**Table 2:** Result of the alignment of the 619 bp sequences of the mtDNA control region obtained in this work (in grey) and haplotypes classified as “haplotype C” based only on a fragment of 394 bp (region marked by the bold black line). Diagnostic sites shown in this table are those obtained aligning all the haplotypes described so far for *P. macrocephalus* at global level (see [20]). All sites identical to those in the reference sequence are indicated as full stops.

			Diagnostic Sites																																	
**Haplotypes**			38	53	57	100	102	104	116	145	179	195	202	203	206	230	233	238	255	267	268	278	281	282	283	284	286	290	300	303	314	319	345	569	603	619
C	001	001	T	T	T	C	A	G	C	C	T	T	A	A	C	A	T	G	A	A	C	C	A	A	G	T	A	G	C	A	G	C	C	G	A	A
C	001	002	.	.	.	.	.	.	.	.	.	.	.	.	.	.	.	.	.	.	.	.	.	.	.	.	.	.	.	.	.	.	.	.	.	G
2330A			.	.	.	.	.	.	.	.	.	.	.	.	.	.	.	.	.	.	.	.	.	.	.	.	.	.	.	.	.	.	.	.	.	G
549			.	.	.	.	.	.	.	.	.	.	.	.	.	.	.	.	.	.	.	.	.	.	.	.	.	.	.	.	.	.	.	.	.	G
400			.	.	.	.	.	.	.	.	.	.	.	.	.	.	.	.	.	.	.	.	.	.	.	.	.	.	.	.	.	.	.	.	.	G
154159			.	.	.	.	.	.	.	.	.	.	.	.	.	.	.	.	.	.	.	.	.	.	.	.	.	.	.	.	.	.	.	.	.	G
456			.	.	.	.	.	.	.	.	.	.	.	.	.	.	.	.	.	.	.	.	.	.	.	.	.	.	.	.	.	.	.	.	.	G
45486			.	.	.	.	.	.	.	.	.	.	.	.	.	.	.	.	.	.	.	.	.	.	.	.	.	.	.	.	.	.	.	.	.	G
463			.	.	.	.	.	.	.	.	.	.	.	.	.	.	.	.	.	.	.	.	.	.	.	.	.	.	.	.	.	.	.	.	.	G
465			.	.	.	.	.	.	.	.	.	.	.	.	.	.	.	.	.	.	.	.	.	.	.	.	.	.	.	.	.	.	.	.	.	G
466			.	.	.	.	.	.	.	.	.	.	.	.	.	.	.	.	.	.	.	.	.	.	.	.	.	.	.	.	.	.	.	.	.	G
467			.	.	.	.	.	.	.	.	.	.	.	.	.	.	.	.	.	.	.	.	.	.	.	.	.	.	.	.	.	.	.	.	.	G
C	002	001	.	.	.	.	.	.	.	.	.	.	.	.	.	.	.	.	.	.	.	.	.	.	.	.	.	.	.	.	.	.	.	T	.	G

**Table 3:** ID number, sex, total body length (TBL) and age of *P. macrocephalus* specimens examined in this study. ND: undetermined sex.

ID	Sex	TBL (m)	Age (ys)
GP-1	M juv	7.2	8
549	F	6.50	24–26
400	F	8.40	21–22
154159	ND juv	6.10	3
456	M juv	8.6	10
45486	M	12.16	40–42
465	F	6.26	5
12988	F	10	38–40

## Data Availability

Newly generated cytogenetic data are available within this manuscript. DNA sequences are available on Genbank (Accession numbers OQ060609–OQ060618).
